# Supplementary material for: Intracellular complexities of acquiring a new enzymatic function revealed by mass-randomisation of active-site residues
Source: eLife. 2020 Nov 13;9:e59081. doi: 10.7554/eLife.59081 (PMC7738182; doi:10.7554/eLife.59081)
Supplement: Supplementary file 1. — (a) Summary of substitutions present in the top 30 chloramphenicol-detoxifying variants generated by multi-site saturation mutagenesis. (b) Kinetic parameters of chloramphenicol reduction and turnover rate of 1,4-benzoquinone for intermediates from the most plausible hypothetical evolutionary trajectories for (A) 36_37 and (B) 20_39. Apparent KM and kcat were calculated using Graphpad 8.0. Kinetic parameters could not be accurately determined for 1,4-benzoquinone, therefore the catalytic rate of 1,4-benzoquinone reduction was measured at a single high concentration of 1,4-benzoquinone (100 µM) with reactions initiated by addition of 250 µM NADPH. All reactions were measured in triplicate and errors are ±1 S.D. In the left-most column, the terminology ‘+’ refers to an enzyme variant that has the same amino acid sequence as the variant in the row above, plus the one additional substitution indicated. For example, ‘+R225V’ describes a variant sharing an identical primary sequence to NfsA, with the additional substitution R225V. *Apparent kcat and KM as determined at 250 µM NADPH. **Measured rates following addition of 250 µM NADPH. ***N.D.=not detectable (change in OD340 <0.1 s−1). (c) Average fold improvement for all NfsA_Ec variants that either retained R225 or contained a R225V/D substitution. To calculate the average fold improvement of variants retaining R225, the fold improvement relative to wild-type NfsA_Ec of all 64 variants retaining R225 was averaged. To calculate the average fold improvement of variants with the R225V or R225D substitutions, the fold improvement relative to wild-type NfsA_Ec of all 64 variants containing either R225V (in 36_37 intermediates) or R225D (in 20_39 intermediates) was averaged. (d) Relative levels of chloramphenicol growth inhibition experienced by E. coli 7NT strains expressing the 50 unique nfsA variants generated by epPCR. Following transformation of E. coli 7NT cells with the epPCR library, plating on LB amended with 10 µM ch [file elife-59081-supp1.docx]

**Supplementary File 1a**

| **NfsA Variant** | **S41** | **L43** | **H215** | **T219** | **K222** | **S224** | **R225** | **F227** |
| --- | --- | --- | --- | --- | --- | --- | --- | --- |
| **36_37** | Y | L | C | Y | V | R | V | G |
| **20_39** | Y | L | N | Y | R | Y | D | H |
| **36_3** | H | S | G | Y | V | H | C | H |
| **19_22** | Y | S | V | F | Q | N | N | R |
| **37_4** | Y | S | G | F | M | H | C | H |
| **35_58** | Y | Y | N | L | E | R | D | H |
| **22_8** | F | C | G | F | R | N | N | H |
| **36_51** | Y | V | N | Y | A | N | G | D |
| **35_45** | Y | V | G | Y | V | H | V | G |
| **21_17** | Y | V | G | Y | A | Y | C | D |
| **36_6** | F | V | F | H | R | D | F | D |
| **21_22** | Y | V | G | F | E | R | N | D |
| **37_5** | Y | C | D | F | L | V | N | G |
| **19_49** | Y | L | D | F | K | I | S | G |
| **22_4** | Y | S | N | F | A | I | N | Y |
| **36_15** | F | L | V | Y | A | H | V | H |
| **37_18** | Y | L | D | L | T | R | N | L |
| **36_22** | Y | C | D | F | K | I | C | D |
| **20_48** | Y | L | D | Y | R | F | N | L |
| **20_5** | Y | C | F | F | Q | H | C | C |
| **36_42** | Y | V | G | Y | T | R | C | D |
| **36_17** | Y | V | D | F | L | V | N | F |
| **35_38** | F | C | H | F | T | S | I | D |
| **36_38** | F | L | D | F | V | R | N | G |
| **36_29** | Y | C | F | F | V | S | C | F |
| **21_49** | Y | C | H | Y | E | N | V | G |
| **36_47** | Y | C | H | Y | A | D | V | S |
| **21_24** | F | S | I | F | E | R | V | G |
| **35_44** | Y | L | I | L | G | L | L | D |
| **36_41** | F | L | Y | F | T | Y | N | Y |

**Supplementary Table S1: Summary of substitutions present in the top 30 chloramphenicol-detoxifying variants generated by multi-site saturation mutagenesis.**

**Supplementary File 1b**

| **A** | **36_37** | ***k_cat_* (s^-1^)** | ***K_M_* (µM)** | ***k_cat_/K_M_***  **(s^-1^/M^-1^)** | **Rate with 100 µM**  ***p-*benzoquinone (s^-1^)** |
| --- | --- | --- | --- | --- | --- |
| **WT NfsA_Ec** | | 0.89 ± 0.03 | 1042 ± 100 | 857 ± 85 | 9.0 ± 0.5 |
| **36_37_3** | | 0.96 ± 0.04 | 690 ± 80 | 1390 ± 170 | 1.0 ± 0.1 |
| **36_37_64** | | 0.15 ± 0.003 | 140 ± 10 | 1090 ± 110 | 0.1 ± 0.03 |
| **36_37_80** | | 0.29 ± 0.02 | 200 ± 50 | 1430 ± 350 | 0 ± 0.03 |
| **36_37_79** | | 0.21 ± 0.006 | 200 ± 20 | 1040 ± 110 | 0 ± 0.03 |
| **36_37_83** | | 0.33 ± 0.008 | 160 ± 10 | 2020 ± 180 | 0.1 ± 0.03 |
| **36_37_122** | | 0.11 ± 0.004 | 24 ± 5 | 4520 ± 970 | -0.1 ± 0.01 |
| **36_37** | | 0.14 ± 0.004 | 130 ± 20 | 1100 ± 150 | -0.1 ± 0.02 |

| **B** | **20_39** | ***k_cat_* (s^-1^)** | ***K_M_* (µM)** | ***k_cat_/K_M_***  **(s^-1^/M^-1^)** | **Rate with 100 µM**  ***p*-benzoquinone (s^-1^)** |
| --- | --- | --- | --- | --- | --- |
| **WT NfsA_Ec** | | 0.89 ± 0.03 | 1042 ± 98 | 857 ± 85 | 9.0 ± 0.5 |
| **20_39_118** | | 0.71 ± 0.04 | 450 ± 70 | 1560 ± 260 | 0.3 ± 0.06 |
| **20_39_85** | | 0.12 ± 0.006 | 140 ± 30 | 840 ± 200 | -0.1 ± 0.03 |
| **20_39_84** | | 0.13 ± 0.004 | 320 ± 30 | 420 ± 40 | 0.0 ± 0.02 |
| **20_39_100** | | 0.27 ± 0.009 | 180 ± 20 | 1560 ± 180 | 0.0 ± 0.04 |
| **20_39_74** | | 0.20 ± 0.006 | 87 ± 13 | 2260 ± 350 | 0.0 ± 0.01 |
| **20_39_124** | | 0.19 ± 0.009 | 240 ± 40 | 790 ± 120 | 0.0 ± 0.01 |
| **20_39** | | 0.16 ± 0.004 | 82 ± 10 | 1920 ± 240 | 0.0 ± 0.02 |

**Supplementary Table S2: Kinetic parameters of chloramphenicol reduction and turnover rate of 1,4-benzoquinone for intermediates from the most plausible hypothetical evolutionary trajectories for (A) 36_37 and (B) 20_39.** Apparent *K_M_* and *k_cat_* were calculated using Graphpad 8.0. Kinetic parameters could not be accurately determined for 1,4*-*benzoquinone, therefore the catalytic rate of 1,4­-benzoquinone reduction was measured at a single high concentration of 1,4-benzoquinone (100 µM) with reactions initiated by addition of 250 µM NADPH. All reactions were measured in triplicate and errors are ± 1 S.D. In the left-most column, the terminology “+” refers to an enzyme variant that has the same amino acid sequence as the variant in the row above, plus the one additional substitution indicated. For example, “+R225V” describes a variant sharing an identical primary sequence to NfsA, with the additional substitution R225V. *****Apparent *k_cat_* and *K_M_* as determined at 250 µM NADPH. ******Measured rates following addition of 250 µM NADPH. *******N.D. = not detectable (change in OD_340_ <0.1 s^-1^).

**Supplementary File 1c**

|  | **Average mean fold improvement of:** | **Average ± SD** |
| --- | --- | --- |
| 36_37 Intermediates | All variants | 2.8 ± 2.4 |
|  | Variants retaining R225 | 1.1 ± 0.6 |
|  | Variants containing R225V substitutions | 4.5 ± 2.2 |
| 20_39 Intermediates | All variants | 2.2 ± 2.2 |
|  | Variants retaining R225 | 0.9 ± 0.2 |
|  | Variants containing R225D substitutions | 3.6 ± 2.4 |

**Supplementary Table S3: Average mean fold improvement for all NfsA_Ec variants that either retained R225 or contained a R225V/D substitution.** To calculate the average fold improvement of variants retaining R225, the fold improvement relative to wild type NfsA_Ec of all 64 variants retaining R225 was averaged. To calculate the average fold improvement of variants with the R225V or R225D substitutions, the fold improvement relative to wild type NfsA_Ec off all 64 variants containing either R225V (in 36_37 intermediates) or R225D (in 20_39 intermediates) was averaged.

**Supplementary File 1d**

**Supplementary Figure S1: Relative levels of chloramphenicol growth inhibition experienced by *E. coli* 7NT strains expressing the 50 unique *nfsA* variants generated by epPCR.** Following transformation of *E. coli* 7NT cells with the epPCR library, plating on LB amended with 10 µM chloramphenicol, and random selection of 60 colonies, 50 unique variants (numbered from ep_1 to ep_50; ep for “error-prone PCR”) were identified by Sanger sequencing of the pUCX inserts. Fresh day cultures of each unique strain were incubated at 30 °C, 200 rpm for 4 hours post-challenge with either a low (7.5 µM), medium (15 µM) or high (30 µM) concentration of chloramphenicol, and percentage growth inhibition was determined by calculating the relative increase in OD_600_ for challenged cultures relative to unchallenged replicates.

**Supplementary File 1e**

|  | **Protein mutations** | | | | **DNA mutations** | | | | | |  |
| --- | --- | --- | --- | --- | --- | --- | --- | --- | --- | --- | --- |
|  | **R225X** | **Additional amino acid changes** | | | **C673X** | **Additional nucleotide changes** | | | | | **# clones** |
| **ep_1** | R225C |  |  |  | C673T |  |  |  |  |  | 5 |
| **ep_2** | R225G | T64S | A119V | E194K | C673G | A190T | T318A | C356T | G580A | C648T | 2 |
| **ep_3** | R225C | N134Y | A190V |  | C673T | G75A | A400T | C569T |  |  | 2 |
| **ep_4** | R225C | G65D |  |  | C673T | G194A | G219T | T480A |  |  | 2 |
| **ep_5** | R225C | E20G |  |  | C673T | A59G |  |  |  |  | 2 |
| **ep_6** | R225C |  |  |  | C673T | G180T | T609A |  |  |  | 2 |
| **ep_7** | R225C | F17Y | A197V |  | C673T | T50A | A60G | G132A | C590T |  | 2 |
| **ep_8** | R225C |  |  |  | C673T | T471C |  |  |  |  | 1 |
| **ep_9** | R225C | E20G | A112G | L142Q | C673T | A59G | C335G | T425A | T459C | C561T | 1 |
| **ep_10** | R225S | Q195L | I221T |  | C673A | T15C | T276C | A584T | T662C |  | 1 |
| **ep_11** | R225C | A74T |  |  | C673T | G220A | A297G | G357T |  |  | 1 |
| **ep_12** | R225C |  |  |  | C673T | C612T |  |  |  |  | 1 |
| **ep_13** | R225G | R50H | T64S |  | C673G | G132A | G149A | A190T | T270C | T498A | 1 |
| **ep_14** | R225C | E178G |  |  | C673T | A533G |  |  |  |  | 1 |
| **ep_15** | R225C | L63M | D214E | K234* | C673T | C187A | T642A |  | A700T |  | 1 |
| **ep_16** | R225S | A25E | I117N | K186R | C673A | C74A | T350A | A557G | T621C |  | 1 |
| **ep_17** | R225S | Q195L | N206Y |  | C673A | C399T | A584T | A616T |  |  | 1 |
| **ep_18** | R225C | W77L | N134S | E198D | C673T | G230T | A401G | C468T | A594T |  | 1 |
| **ep_19** | R225C | Q147R |  |  | C673T | C156G | C255T | A440G |  |  | 1 |
| **ep_20** | R225C | I117N |  |  | C673T | T350A | C351T |  |  |  | 1 |
| **ep_21** | R225C |  |  |  | C673T | G213A |  |  |  |  | 1 |
| **ep_22** | R225C |  |  |  | C673T | C192T |  |  |  |  | 1 |
| **ep_23** | R225C | Q195H |  |  | C673T | T24A | A585T | G705A |  |  | 1 |
| **ep_24** | R225S | Q26K | D162E |  | C673A | C76A | G87A | G357T | T486A | C624A | 1 |
| **ep_25** | R225S | E24G | T202A |  | C673A | A71G | A604G |  |  |  | 1 |
| **ep_26** | R225C | I31T |  |  | C673T | T92C |  |  |  |  | 1 |
| **ep_27** | R225C | E58G | A81G | G131A | C673T | A173G | C242G | C243T | G392C | G562T | 1 |
| **ep_28** | R225C | T64S |  |  | C673T | C191G |  |  |  |  | 1 |
| **ep_29** | R225C | S180C |  |  | C673T | G306A | T507A | A538T |  |  | 1 |
| **ep_30** | R225C | R27C | V176A | T239R | C673T | C79T | T117C | A174G | T527C | C716G | 1 |
| **ep_31** | R225C |  |  |  | C673T | T523C |  |  |  |  | 1 |
| **ep_32** | R225C | H16R | K68N | Q72L | C673T | A47G | A204T | A215T | A534C | A654G | 1 |
| **ep_33** | R225C | A29S | P168L | R225C | C673T | G85T | C309A | C503T | C579T |  | 1 |
| **ep_34** | R225C | T64A | K141R | P168Q | C673T | A190G | C351T | A422G | C503A |  | 1 |
| **ep_35** | R225C | H16Q |  |  | C673T | T48A | G186A |  |  |  | 1 |
| **ep_36** | R225C | K141R |  |  | C673T | A422G |  |  |  |  | 1 |
| **ep_37** | R225G |  |  |  | C673G | C285T |  |  |  |  | 1 |
| **ep_38** | R225G |  | P226S |  | C673G | A174G | C561T | C624T | C676T |  | 1 |
| **ep_39** | R225C | A74T |  |  | C673T | A72G | G213C | G220A |  |  | 1 |
| **ep_40** | R225C |  |  |  | C673T | T279A | T321C | G396A | C448T | G501A | 1 |
| **ep_41** | R225C | A25V |  |  | C673T | C74T |  |  |  |  | 1 |
| **ep_42** | R225C | G123E |  |  | C673T | G368A |  |  |  |  | 1 |
| **ep_43** | R225S | E99D |  |  | C673A | A297C |  |  |  |  | 1 |
| **ep_44** | R225G |  |  |  | C673G | A18G | T42A |  |  |  | 1 |
| **ep_45** | R225C | A119V |  |  | C673T | G78A | A210G | C356T |  |  | 1 |
| **ep_46** | R225C | G10C | L56F | G66E | C673T | G28T | A168T | G197A | C309A | C387T | 1 |
| **ep_47** | R225C | A197V |  |  | C673T | A201G | C590T |  |  |  | 1 |
| **ep_48** | R225G | L201F |  |  | C673G | C601T |  |  |  |  | 1 |
| **ep_49** | R225C |  |  |  | C673T | T144A |  |  |  |  | 1 |
| **ep_50** | R225C | E59K |  |  | C673T | C63T | G175A | G717C |  |  | 1 |

**Supplementary Table S4: Summary of all encoded amino acid and nucleotide substitutions identified in the 50 unique *nfsA* variants obtained from the epPCR library following chloramphenicol selection.**

**Supplementary File 1f**

|  | **Amino acid changes** | | | | | | **Nucleotide changes** | | | | | | |
| --- | --- | --- | --- | --- | --- | --- | --- | --- | --- | --- | --- | --- | --- |
| Rand_1 | N207S | Q182H |  |  |  |  | A620G | A546C |  |  |  |  |  |
| Rand_2 | A112T | E198G |  |  |  |  | G334A | A593G |  |  |  |  |  |
| Rand_3 | V105D | L201H |  |  |  |  | T314A | G324T | T602A |  |  |  |  |
| Rand_4 | E75G | D82N |  |  |  |  | A224G |  | G244A | T609C |  |  |  |
| Rand_5 |  |  |  |  |  |  | G627T |  |  |  |  |  |  |
| Rand_6 | A29V | T52I | F83I | W212C |  |  | C86T | T126C | C155T | T247A | G636T |  |  |
| Rand_7 | P146S |  |  |  |  |  | G213T | C436T | C624T |  |  |  |  |
| Rand_8 | E6V | S13Y | D107N | R209Q | P226T |  | A17T | C38A | T105A | G319A | G626A | C651T | C676A |
| Rand_9 |  |  |  |  |  |  | T126C | C156A |  |  |  |  |  |
| Rand_10 | A71T | D92V | N179Y |  |  |  | G211A | A275T | A535T |  |  |  |  |
| Rand_11 | R50H | D162N | N207D | Y207N |  |  | G149A | T318G | G484A | G570T | A619G | T619A |  |
| Rand_12 | G10D | A119V |  |  |  |  | G29A | C356T | G372A |  |  |  |  |
| Rand_13 |  |  |  |  |  |  |  |  |  |  |  |  |  |
| Rand_14 | A74V | R85H | E194A | I228V |  |  | C221T | G254A | A354T | A581C | A682G |  |  |
| Rand_15 | P160H | A188E |  |  |  |  | C315A | A381G | C479A | C563A |  |  |  |
| Rand_16 | P160H | A188E |  |  |  |  | C315A | A381G | C479A | C563A |  |  |  |
| Rand_17 |  |  |  |  |  |  | ΔG198 |  |  |  |  |  |  |
| Rand_18 |  |  |  |  |  |  | A654G |  |  |  |  |  |  |
| Rand_19 | P21L | L116I | R217H |  |  |  | C62T | T346A | C606T | G650A |  |  |  |
| Rand_20 | A16R | D19Y | H69Y | F83S | A118V | D165E | A47G | G55T | C205T | T248C | C353T | T495A |  |

**Supplementary Table S5: Summary of all encoded amino acid and nucleotide substitutions identified in 20 randomly-chosen *nfsA* variants obtained from the epPCR library in the absence of chloramphenicol selection.**
